# ANAID‐ICH nomogram for predicting unfavorable outcome after intracerebral hemorrhage

**DOI:** 10.1111/cns.13941

**Published:** 2022-08-24

**Authors:** Jiawen Li, Dong Luo, Feifei Peng, Qi Kong, Huawei Liu, Meiyuan Chen, Lusha Tong, Feng Gao

**Affiliations:** ^1^ Department of Neurology, The Second Affiliated Hospital Zhejiang University School of Medicine Hangzhou China; ^2^ Department of Neurology The Affiliated Hospital of Hangzhou Normal University Hangzhou China

**Keywords:** cerebral hemorrhage, magnetic resonance imaging, nomogram, prognosis

## Abstract

**Objective:**

Diffusion‐weighted imaging lesions (DWILs) are associated with unfavorable outcome in intracerebral hemorrhage (ICH). We proposed a novel predictive nomogram incorporating DWILs.

**Methods:**

A total of 738 patients with primary ICH in a tertiary hospital were prospectively enrolled as a training cohort. DWILs were defined as remote focal hyperintensities on DWI corresponding to low intensities on apparent diffusion coefficient images and remote from the focal hematoma. The outcome of interest was modified Rankin Scale scores of 4–6 at 90 days after onset. Multivariate logistic regression was used to construct a nomogram. Model performance was tested in the training cohort and externally validated with respect to discrimination, calibration, and clinical usefulness in another institute. Additionally, the nomogram was compared with the ICH score in terms of predictive ability.

**Results:**

Overall, 153 (20.73%) and 23 (15.54%) patients developed an unfavorable outcome in the training and validation cohorts, respectively. The multivariate analysis revealed that age, National Institutes of Health Stroke Scale (NIHSS) score, anemia, infratentorial location, presence of DWILs, and prior ICH were associated with unfavorable outcome. Our ANAID‐ICH nomogram was constructed according to the aforementioned variables; the area under the receiver operating characteristic curve was 0.842 and 0.831 in the training and validation sets, respectively. With regard to the 90‐day outcome, the nomogram showed a significantly higher predictive value than the ICH score in both cohorts.

**Conclusions:**

The ANAID‐ICH nomogram comprising age, NIHSS score, anemia, infratentorial location, presence of DWILs, and prior ICH may facilitate the identification of patients at higher risk for an unfavorable outcome.

## Graphical abstract

Using data from 738 patients with primary ICH, this study identifies 6 predictors for 90‐day unfavorable outcomes, including age, NIHSS score, anemia, infratentorial location, presence of DWILs, and prior ICH. A novel nomogram based on these factors shows remarkable discrimination and calibration in the training and validation sets, respectively. It may be helpful for clinical decision‐making and patients surveillance.

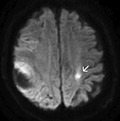

## 1 INTRODUCTION

Intracerebral hemorrhage (ICH) is a devastating disease with higher mortality and morbidity rates than any other form of stroke.^1^ Approximately, 40% of patients has been estimated to die at 1 year, and most survivors suffer from disability.^2^, ^3^ The often dismal outcome highlights the need for establishing prognostic models to aid clinicians in selecting treatments and monitoring patients. The ICH score introduced by Hemphill et al.,^4^ comprises age, hematoma volume, Glasgow Coma Scale (GCS) score, intraventricular hemorrhage (IVH), and infratentorial location and has been broadly applied in clinical ICH studies.^5^ While the ICH score had been shown to have great value in discriminating the risk of mortality,^6^ the results varied in terms of functional outcomes.^7^, ^8^, ^9^ This discrepancy might be partly because the ICH score represented the effect of hematoma; however, the outcome was also determined by the brain status globally in the ICH setting.^10^, ^11^

Diffusion‐weighted imaging lesions (DWILs) are found in approximately 41% of patients with ICH.^12^, ^13^ These lesions were previously observed to be related to imaging markers of cerebral small vessel disease, such as cerebral microbleeds, white matter hyperintensity, and enlarged perivascular spaces, suggesting that global microangiopathy predates ICH.^13^, ^14^, ^15^, ^16^, ^17^, ^18^, ^19^ A large‐scale pooled analysis of 1752 patients across four trials showed that patients with DWILs had an elevated risk of unfavorable outcome at 90 days after ICH.^19^ Indeed, cumulative evidence supports the unfavorable effects of DWILs on morbidity and mortality following ICH. We hypothesized that the inclusion of DWILs in a prognostic model might improve the prediction efficiency.

The nomogram has been extensively used as a predictive model in surgery, cancer, and other specialties.^20^, ^21^ It incorporates critical variables in prognosis and provides a continuum risk estimation of an individual patient. The present study aimed to (i) develop a novel nomogram based on clinical factors and DWILs with regard to 90‐day functional outcome, (ii) investigate the predictive power of our nomogram and ICH score, and (iii) validate our nomogram in another institute.

## 2 METHODS

### 2.1 Study population

**FIGURE 1:**
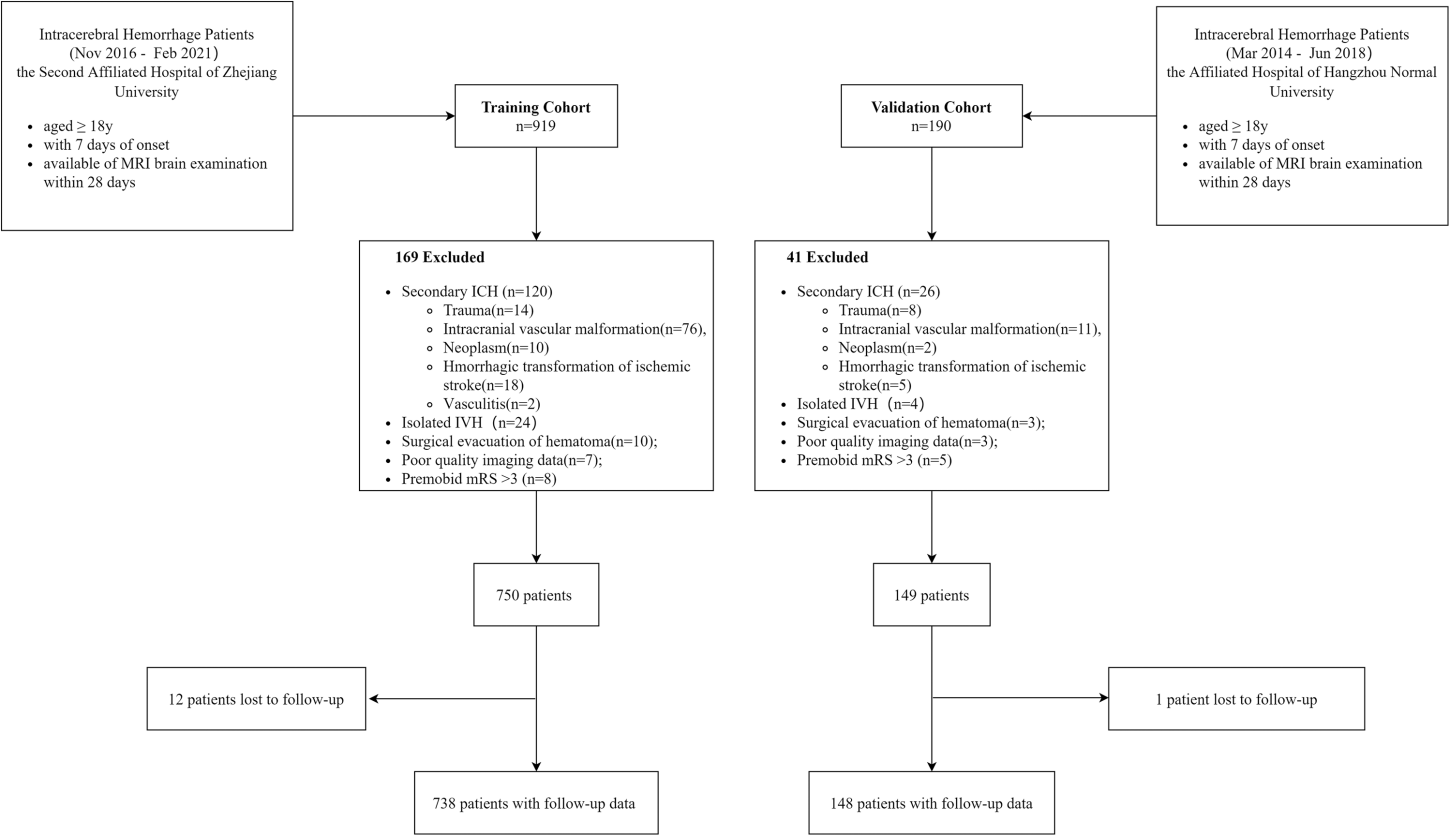
Selection flowchart of included and excluded patients.

Patients with ICH from the Department of Neurology at the Second Affiliated Hospital of Zhejiang University from November 2016 to February 2021 were prospectively enrolled. Eligible patients who met the following criteria were recruited as the training cohort: (i) age ≥18 years; (ii) diagnosis of ICH within 7 days from symptom onset or the last time seen well; and (iii) magnetic resonance imaging (MRI) performed within 28 days after ICH. Patients with any of the following characteristics were excluded: (i) hemorrhage due to trauma, intracranial vascular malformation, neoplasm, or any other presumed cause of secondary ICH; (ii) isolated IVH; (iii) any type of surgical hematoma evacuation; (iv) poor‐quality imaging data; (v) baseline modified Rankin Scale (mRS) score of >3; or (vi) loss to follow‐up (Figure 1).

An independent cohort of patients was enrolled at the Affiliated Hospital of Hangzhou Normal University from March 2014 to June 2018. These patients were screened using the same inclusion and exclusion criteria to serve as an external validation cohort (Figure 1).

### 2.2 Data availability

We collected data on age, sex, and clinical variables, including systolic blood pressure, diastolic blood pressure, time from ictus to emergency, National Institutes of Health Stroke Scale (NIHSS) score, and GCS score at initial presentation. Using a standard assessment form, trained physicians obtained data on the functional status prior to onset, comorbidities (e.g., hypertension, diabetes mellitus, atrial fibrillation, and prior stroke), and pre‐hospital use of antiplatelet and anticoagulation agents. The following laboratory parameters were retrieved from medical records: hemoglobin, platelet count, international normalized ratio (INR), fasting blood glucose, low‐density lipoprotein (LDL), and creatine. Hemoglobin, platelet count, and INR were measured on admission, whereas fasting blood glucose, LDL, and creatine were measured on the next morning after admission. Anemia was defined as hemoglobin concentration <120 g/L in women and <130 g/L in men.^22^

### 2.3 Imaging acquisition and analysis

Non‐contrast computed tomography (NCCT) was performed using multidetector row scanners with 4.8‐ or 5‐mm slice thickness axial reconstruction, tube potential of 120–130 kVp, and tube current of 100–300 mA in accordance with the local NCCT protocol at each site. The ICH location was assessed on baseline NCCT scans and classified into lobar (involvement of the cortical surface or juxtacortical regions), deep (involvement of the thalamus, basal ganglia, or internal capsule), and infratentorial (involvement of the brain stem and cerebellum). Volumetric assessment was completed using a semiautomated planimetric method with the ITK‐SNAP software (University of Pennsylvania, Philadelphia, USA; www.itksnap.org). IVH was defined as an intraventricular hyperdense image that was not attributable to the choroid plexus or calcification and was not included in the hematoma volume.

MRI was performed on a 1.5‐Tesla (Sonata, Siemens Healthcare GmbH, Erlangen, Germany) or 3.0‐Tesla scanner (Signa HDxt, GE Healthcare, Milwaukee, WI, USA) with a standardized protocol consisting of axial T1‐weighted, T2‐weighted, T2‐FLAIR, DWI, and apparent diffusion coefficient (ADC) sequences at our site. Axial DWI sequences were acquired on a 1.5‐Tesla scanner [repetition time (TR) = 3100 ms, echo time (TE) = 84 ms, b = 0/1000 s/mm^2^, 6‐mm slice thickness, 0.5‐mm gap, field of view (FOV) = 230 mm] or a 3.0‐Tesla scanner (TR = 5200 ms, TE = 75 ms, b = 0/1000 s/mm^2^, 6‐mm slice thickness, 0.5‐mm gap, FOV = 240 mm). In the validation cohort, patients underwent MRI scans on a 1.5‐Tesla scanner (Avanto, Siemens), and DWI sequences were performed with the following parameters: TR = 4000 ms, TE = 102 ms, b = 0/1000 s/mm^2^, 5.0‐mm slice thickness, 1.5‐mm gap, and FOV = 230 mm.

DWILs were defined as focal hyperintensities on DWI corresponding to low intensities on ADC images and remote from the focal hematoma (>20 mm).^17^ All rules were formulated prior to the current study. Two experienced raters (Q. Kong and H. Liu), who were blinded to the clinical and outcome data, reviewed all images separately to determine the presence, number, and distribution of DWILs. Using a subset of 30 representative cases from our training cohort, the inter‐rater reliability for DWIL assessment between the two trained raters was determined to be excellent (kappa = 0.86).

### 2.4 Follow‐up

Patients were followed up during face‐to‐face clinic visits or via telephone interviews at 90 days after ICH. We recorded the mRS scores as the neurological outcome. A favorable outcome was defined as mRS scores of 0–3, whereas an unfavorable outcome was defined as mRS scores of 4–6.

### 2.5 Statistical analysis

Categorical variables are summarized as *n* (%) and were compared using the χ^2^ test or Fisher's exact test, as appropriate. Continuous variables are expressed as mean ± standard deviation or as median (interquartile range [IQR]) based on their distribution, as evaluated using the Shapiro–Wilk test. Student's *t*‐test or Mann–Whitney *U* test was used to compare the continuous variables. The inter‐rater agreement for DWILs was calculated for a sample of 30 patients using κ coefficient.

To develop a nomogram with good calibration and discrimination, a model was constructed in our training set and was subsequently validated in another dataset. Variables with *p* < 0.1 in the univariate regression analysis and those identified in previously published articles were included in the multivariate logistic regression models to identify the independent risk factors for the 90‐day outcome. We used backward elimination procedures to arrive at a minimal model including only variables at *p* < 0.05. Potential multicollinearity was evaluated using variance inflation factor (VIF), and an arithmetic square root of VIF ≤2 was considered as non‐collinearity. A nomogram was formulated based on all independent predictors that predict poor outcome in the final model.

The discriminative ability of the nomogram was summarized by calculating the area under the receiver operating characteristic curve (AUC‐ROC). Calibration was measured using the Hosmer–Lemeshow test and a calibration plot with a bootstrap of 1000 resamples. To assess the performance of the nomogram in the external validation cohort, the established nomogram was then applied to predict the probability of an unfavorable outcome, and the AUC‐ROC, calibration curve, and Hosmer–Lemeshow test were applied. Pairwise comparison of the AUC‐ROC was conducted to compare the nomogram with the ICH score in each cohort. Categorical net reclassification improvement (NRI) and integrated discrimination improvement (IDI) were used to determine the extent to which the predictive power of the nomogram improved than the ICH score system. Decision curve analysis (DCA) was carried out to assess the clinical usefulness of our nomogram and ICH score by quantifying the net benefit across different threshold probabilities in the training and validation cohorts.^23^

All statistical analyses were performed using R software version 4.0.1 (http://www.Rproject.org). The nomogram and calibration plot were constructed using the “rms” package; the NRI and IDI were calculated using the “PredictABEL” package; and the DCA was established using the “rmda” package. A two‐tailed value of *p* < 0.05 was considered to be indicative of statistical significance.

## 3 RESULTS

**FIGURE 2:**
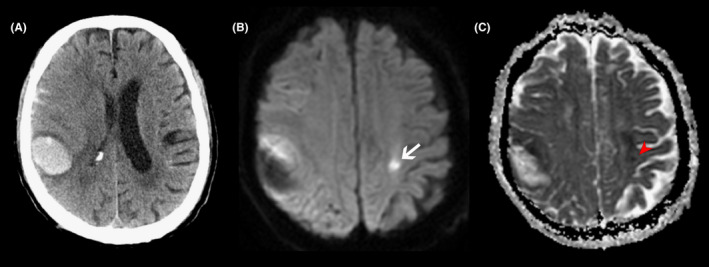
Representative MRI of diffusion‐weighted imaging lesions (DWILs) in patients with acute intracerebral hemorrhage. In a patient with lobar hemorrhage (A), the lesion (white arrow) appears as hyperintensity on diffusion‐weighted imaging (B) with corresponding hypointensity signal (red arrow) on apparent diffusion coefficient map (C).

Figure 1 presents the flowchart of patient inclusion. A total of 738 eligible patients were included in the training cohort. Of these, 66.94% were men, and the median age was 62 (IQR, [52, 70]) years. In the training cohort, we identified 168 DWILs in 131 individuals: 25 patients had at least two lesions; the rest had a single DWI lesion. All lesions were round or ovoid and were at least 20 mm distant from the closet hemorrhage. Representative examples of DWI lesions are shown in Figure 2. DWILs were most common found in lobar cortical–subcortical areas (*n* = 132 [78.6%]) especially the frontal lobes (*n* = 66 [39.4%]). Of the remainder, 28 (16.7%) were deep hemispheric and 8 (4.8%) infratentorial. Most DWILs (*n* = 97 [57.7%]) were ipsilateral to the ICH. A total of 153 (20.73%) patients developed an unfavorable outcome at 90 days after ICH. Table 1 summarizes the baseline characteristics of the patients grouped according to outcome.

**TABLE 1:** Baseline characteristics of all patients and patients grouped by 90‐day outcome in the training cohort

Characteristic	Overall	mRS 0–3	mRS 4–6	*p*‐Value
	*N* = 738^a^	*N* = 585	*N* = 153	
Age, years, median (IQR)	62 (52, 70)	61 (51, 69)	66 (57, 77)	<0.001**
Male gender, *n* (%)	494 (66.94%)	400 (68.38%)	94 (61.44%)	0.104
Medical history, *n* (%)				
Hypertension	567 (76.83%)	456 (77.95%)	111 (72.55%)	0.159
Diabetes mellitus	130 (17.62%)	105 (17.95%)	25 (16.34%)	0.642
Atrial fibrillation	25 (3.39%)	21 (3.59%)	4 (2.61%)	0.553
Prior ICH	51 (6.91%)	35 (5.98%)	16 (10.46%)	0.052
Prior AIS/TIA	66 (8.94%)	47 (8.03%)	19 (12.42%)	0.091
Antiplatelet	69 (9.35%)	49 (8.38%)	20 (13.07%)	0.076
Anticoagulation	11 (1.49%)	8 (1.37%)	3 (1.96%)	0.706
Baseline clinical assessment, median (IQR)				
Onset to emergency, days	0.50 (0.25, 1.00)	0.50 (0.25, 1.25)	0.25 (0.25, 1.00)	0.060
GCS score	15 (14, 15)	15 (14, 15)	14 (11, 15)	<0.001**
NIHSS score	4 (2, 10)	3 (1, 7)	11 (7, 15)	<0.001
SBP, mmHg	159 (144, 178)	158 (145, 178)	160 (143, 179)	0.602
DBP, mmHg	91 (80, 101)	91 (81, 101)	87 (79, 102)	0.252
MAP, mmHg	114 (103, 125)	114 (103, 125)	113 (101, 127)	0.794
Laboratory values, median (IQR)				
Hemoglobin, g/L	142 (130, 152)	144 (132, 153)	135 (125, 147)	<0.001**
Anemia, *n* (%)	131 (17.75%)	90 (15.38%)	41 (26.80%)	0.001**
Platelet, 10^9^/L	197 (162, 238)	197 (162, 238)	198 (159, 235)	0.685
INR	1.00 (0.96, 1.05)	1.00 (0.96, 1.05)	1.01 (0.97, 1.07)	0.016*
FBG, mmol/L	5.71 (5.02, 6.82)	5.61 (4.95, 6.68)	6.22 (5.24, 7.55)	<0.001**
Creatine, μmol/L	61 (51, 73)	62 (52, 74)	60 (49, 71)	0.136
LDL ≤1.8, *n* (%)	129 (17.67%)	102 (17.62%)	27 (17.88%)	0.940
Radiological variables				
ICH volume, ml, median (IQR)	9.00 (3.36, 17.69)	8.12 (3.00, 16.17)	12.32 (6.00, 22.05)	<0.001**
ICH location, *n* (%)^b^				
Lobar	164 (22.22%)	130 (22.22%)	34 (22.22%)	1.000
Deep	490 (66.40%)	386 (65.98%)	104 (67.97%)	0.643
Infratentorial	108 (14.63%)	83 (14.19%)	25 (16.34%)	0.503
IVH, *n* (%)	215 (29.13%)	157 (26.84%)	58 (37.91%)	0.007*
Onset to MRI, days, median (IQR)	6 (4, 7)	6 (4, 7)	6 (4, 8)	0.022*
Presence of DWILs, *n* (%)	131 (17.75%)	84 (14.36%)	47 (30.72%)	<0.001**

Abbreviations: AIS, acute ischemic stroke; DBP, diastolic blood pressure; DWILs, diffusion‐weighted imaging lesions; FBG, fasting blood glucose; GCS, Glasgow coma scale; ICH, intracerebral hemorrhage; INR, international normalized ratio; IQR, interquartile range; IVH, intraventricular extension hemorrhage; LDL, low‐density lipoprotein; MAP, mean arterial pressure; NIHSS, National Institutes of Health Stroke Scale; SBP, systolic blood pressure; TIA, transient ischemic attack.

^a^ 12 patients without follow‐up data were excluded from the analysis.

^b^ In 25 patients with simultaneous multiple intracerebral hemorrhage, each hematoma was recorded.

*p < 0.05.

**p < 0.01.

The results of the univariate and multivariate logistic analyses are presented in Table 2. The univariate analysis showed that age, time from ictus to emergency, NIHSS score, GCS score, prior ICH, prior acute ischemic stroke/transient ischemic attack, prior antiplatelet agent use, fasting blood glucose level, anemia, baseline ICH volume, IVH extension, and presence of DWILs were associated with an unfavorable outcome (*p* < 0.1). We included these factors and infratentorial location in binary logistic regression models. Based on backward stepwise elimination, age (odds ratio [OR], 1.036; 95% CI [confidence interval], 1.018–1.054; *p* < 0.001), NIHSS score (OR, 1.220; 95% CI, 1.173–1.269; *p* < 0.001), prior ICH (OR, 2.330; 95% CI, 1.139–4.768; *p* = 0.021), anemia (OR, 1.788; 95% CI, 1.071–2.984; *p* = 0.026), infratentorial location (OR, 2.438; 95% CI, 1.365–4.356; *p* = 0.003), and presence of DWILs (OR, 2.819; 95% CI, 1.721–4.618; *p* < 0.001) were identified as independent predictors of 90‐day unfavorable outcome in adjusted model. No significant collinearity was observed for any variables included in the model.

**TABLE 2:** Univariate and multivariate analysis of 90‐day unfavorable outcome (mRS >3) in the training cohort

	Unadjusted		Adjusted model	
Characteristic	OR (95% CI)	*p*‐Value	OR (95% CI)	*p*‐Value
Age, years	1.039 (1.024–1.054)	<0.001	1.044 (1.025–1.063)	<0.001
Onset to emergency, days	0.871 (0.764–0.992)	0.037		
GCS score	0.778 (0.719–0.841)	<0.001		
NIHSS score	1.197 (1.155–1.239)	<0.001	1.220 (1.173–1.269)	<0.001
Prior ICH	1.835 (0.987–3.413)	0.055	2.452 (1.187–5.064)	0.015
Prior AIS/TIA	1.623 (0.922–2.857)	0.093		
Antiplatelet	1.645 (0.946–2.862)	0.078		
Anemia	2.013 (1.320–3.072)	0.001	1.735 (1.035–2.910)	0.037
FBG, mmol/L	1.145 (1.066–1.230)	<0.001		
ICH volume, ml	1.027 (1.015–1.039)	<0.001		
IVH	1.664 (1.145–2.420)	0.008		
Infratentorial location	1.181 (0.726–1.923)	0.503	2.797 (1.514–5.165)	0.001
Presence of DWILs	2.645 (1.748–4.001)	<0.001	2.546 (1.531–4.232)	<0.001
ICH Score	2.028 (1.658–2.480)	<0.001		

Abbreviations: 95% CI, 95% confidence interval; AIS, acute ischemic stroke; DWILs, diffusion‐weighted imaging lesions; FBG, fasting blood glucose; GCS, Glasgow coma scale; ICH, intracerebral hemorrhage; IVH, intraventricular extension hemorrhage; NIHSS, National Institutes of Health Stroke Scale; OR, odds ratio; TIA, transient ischemic attack.

**FIGURE 3:**
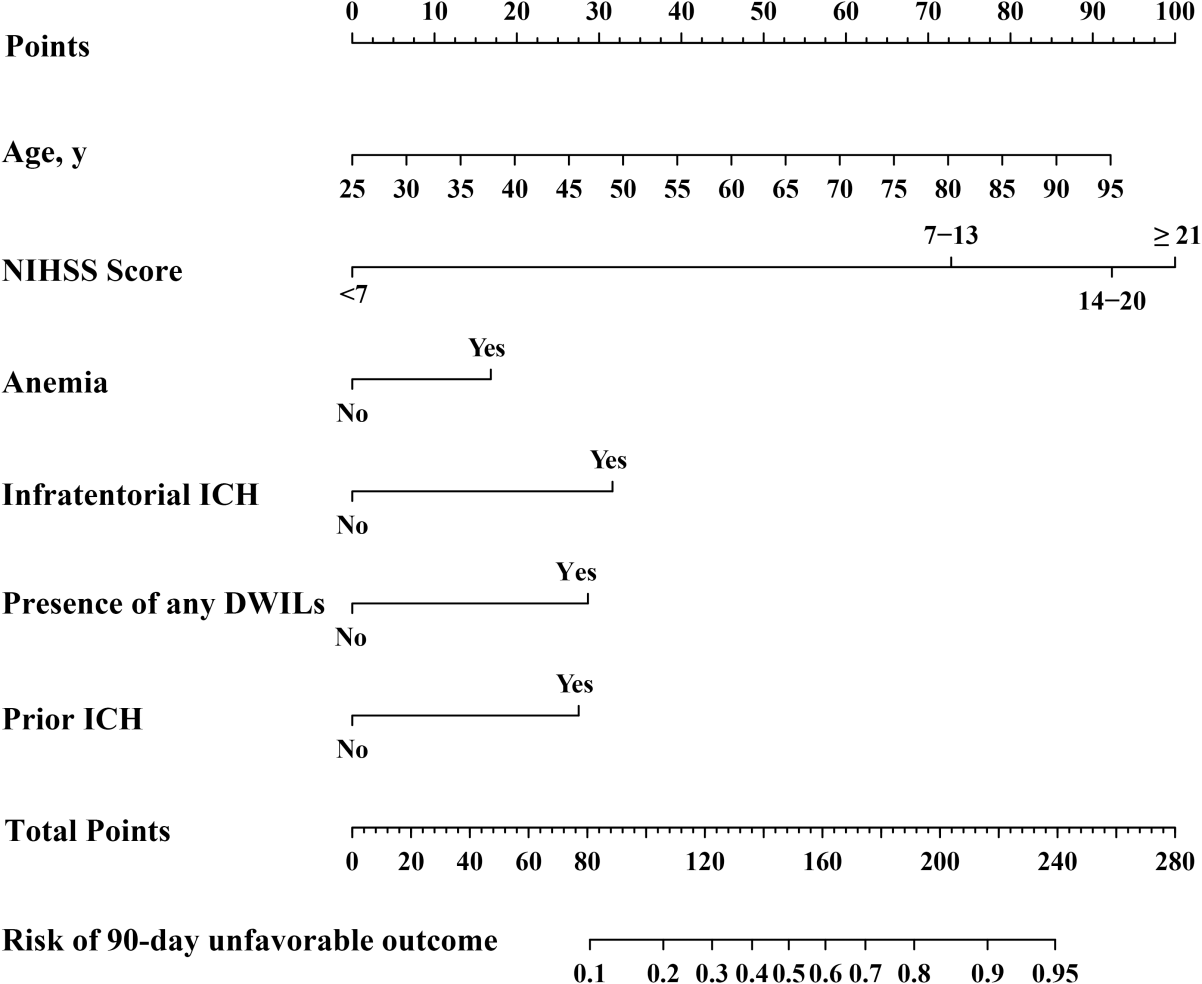
ANAID‐ICH nomogram for predicting the probability of unfavorable outcome at 90 days after ICH. NIHSS indicates the National Institutes of Health Stroke Scale; DWILs, difussion‐weighted imaging lesions; ICH, intracerebral hemorrhage

**FIGURE 4:**
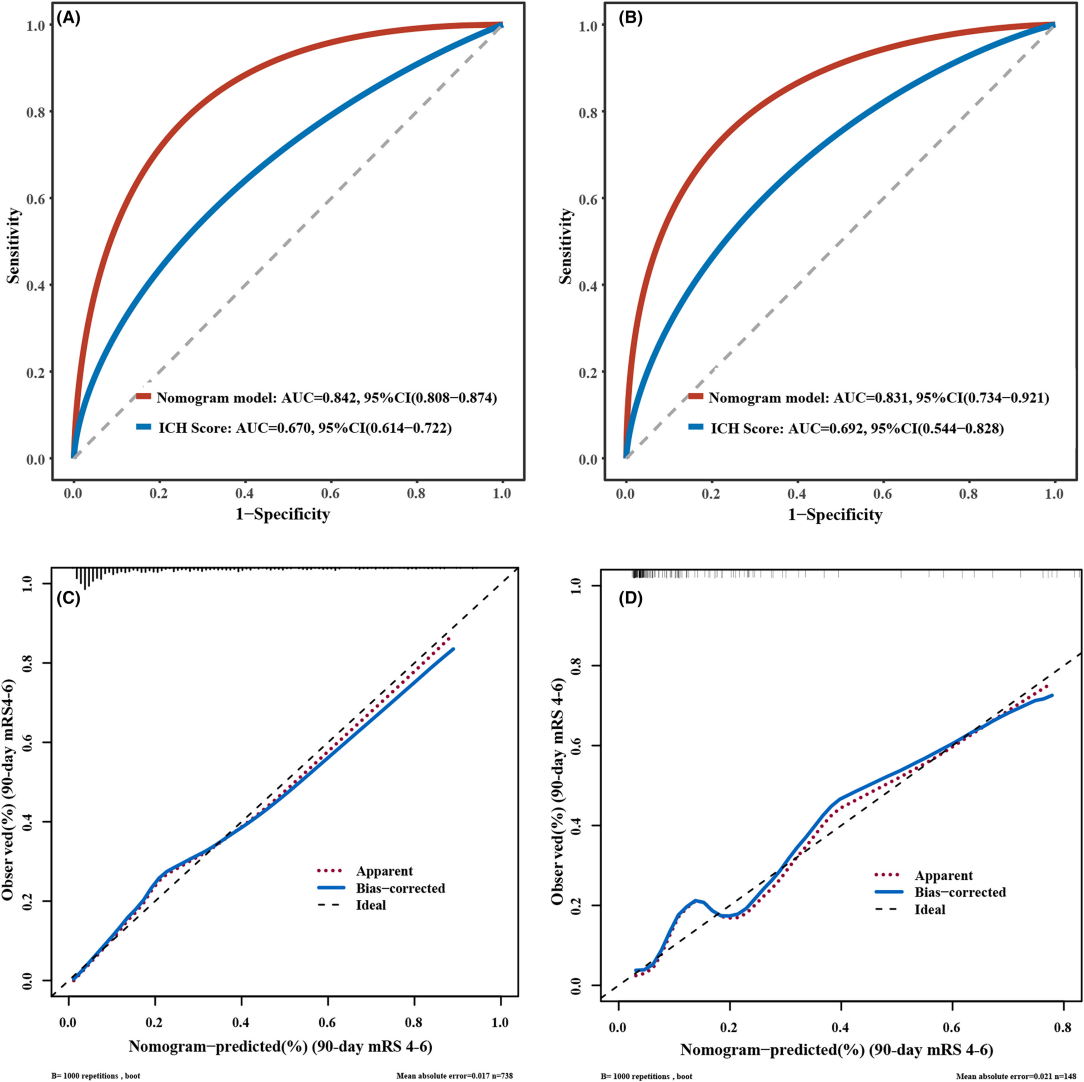
Receiver operating characteristic curves detect the discriminative ability of nomogram in the training (A) and validation cohort (B). Calibration plot for nomogram‐predicted probability of 90‐day unfavorable outcome in the training (C) and validation cohort (D).

Our nomogram model named ANAID‐ICH nomogram was established with age, NIHSS score (categorized as NIHSS <7, 7–13, 14–20, and ≥21), anemia, infratentorial location, presence of DWILs, and prior ICH (Figure 3). The AUC‐ROC of the nomogram for predicting the 90‐day unfavorable outcome was 0.842 (95% CI, 0.808–0.874), indicating an outstanding model discriminative ability (Figure 4A). Furthermore, the calibration plots revealed remarkable predictive accuracy between nomogram prediction and actual probability in the training cohort (Figure 4C). Similarly, the Hosmer–Lemeshow test yielded a non‐significant statistic (*p* = 0.13), indicating acceptable goodness‐of‐fit. In order to further test the developed nomogram, 149 additional patients from another institution were enrolled to serve as the validation cohort. The baseline characteristics of the training and validation cohorts are summarized in Table S1. No significant difference in outcome was noted (20.73% vs. 15.54%, *p* = 0.15). The ANAID‐ICH nomogram showed an AUC‐ROC of 0.831 (95% CI, 0.734–0.921) for the estimation of unfavorable outcome in the validation cohort (Figure 4B). Additionally, the calibration plot confirmed the good calibration of the ANAID‐ICH nomogram (Figure 4D).

**FIGURE 5:**
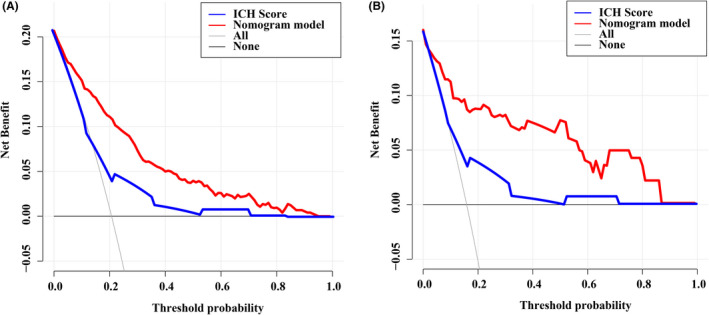
Decision curve analysis of our nomogram and ICH score in the training (A) and validation cohorts (B).

We further compared the AUC‐ROC of the ANAID‐ICH nomogram with that of the ICH score model. The results indicated that the predictive power of our nomogram was superior to that of the ICH score in the training cohort (0.842 vs. 0.669, *p* < 0.001; Figure 4A). Similar significant associations were also observed in the validation cohort (0.831 vs. 0.692, *p* = 0.03; Figure 4B). In terms of reclassification ability, our nomogram showed significantly improved prediction performance than that of the ICH score model in training cohort (categorical NRI: 0.450 [0.414–0.583], *p* < 0.001; IDI: 0.297 [0.257–0.337], *p* < 0.001) and validation cohort (categorical NRI: 0.886 [0.702–1.070], *p* < 0.001; IDI: 0.883 [0.781–0.985], *p* < 0.001; Table 3). Finally, the DCA revealed the clinical usefulness of the models. When the threshold probabilities ranged from 10% to 90% in the training cohort and from 10% to 83% in the validation cohort, the ANAID‐ICH nomogram model showed a positive net benefit, which was clearly better to the ICH score in both the training (Figure 5A) and validation (Figure 5B) cohort.

**TABLE 3:** Comparison of the ANAID‐ICH nomogram model and the ICH Score

	Training cohort			Validation cohort		
	ICH Score	Nomogram	*p*‐Value	ICH score	Nomogram	*p*‐Value
AUC (95% CI)	0.669 (0.614–0.722)	0.842 (0.808–0.874)	<0.001	0.692 (0.544–0.828)	0.831 (0.734–0.921)	0.031
Categorical NRI (95% CI)	Reference	0.450 (0.414–0.583)	<0.001	Reference	0.886 (0.702–1.070)	<0.001
IDI (95% CI)	Reference	0.297 (0.257–0.337)	<0.001	Reference	0.883 (0.781–0.985)	<0.001

Abbreviations: AUC, area under curve; CI, confidence interval; ICH, intracerebral hemorrhage; IDI, integrated discrimination improvement; NRI, net reclassification improvement.

## 4 DISCUSSION

In the present study, we confirmed that DWILs were independently associated with an unfavorable outcome in 738 patients with primary ICH. We then developed and validated a novel nomogram (i.e., the ANAID‐ICH nomogram) consisting of age, NIHSS score, anemia, infratentorial location, presence of DWILs, and prior ICH to predict the 90‐day unfavorable outcome. Our nomogram performed well in both the training and validation cohorts in terms of discrimination and calibration. Furthermore, it was more effective in predicting worse outcome than the ICH score.

A growing body of evidence has shown the unfavorable effects of DWILs on morbidity and mortality after ICH. A pooled analysis of 1752 patients across four trials reported that DWILs occurred in 31.3% of patients with ICH and were independently associated with worse outcome at 90 days.^19^ Notably, several studies included patients who underwent surgical hematoma evacuation, which might have a significant impact on the functional outcome.^16^, ^19^, ^24^ The strength of our study is that we excluded those patients and were able to better isolate the influence of DWILs on the outcome. Nonetheless, the pathogenic mechanisms underlying DWILs remain unclear. Besides the primary brain injury, the parenchymal hematoma triggers a series of adverse events causing secondary injury, which plays an essential role in neurological impairment.^25^, ^26^, ^27^ According to the body of literature to date, the presence of DWILs may indicate advanced cerebral small vessel disease burden, which suggests severe endothelial failure,^28^, ^29^ reduced connectivity of the brain,^30^ and susceptibility to the cytokines and prothrombotic milieu generated by a hematoma,^31^, ^32^, ^33^ all of which may affect post‐ICH recovery.

In our study, several established risk factors, such as age, higher baseline NIHSS score, infratentorial location, and prior ICH, remained relevant to the unfavorable outcome.^34^ In addition, our results support that anemia is an independent predictor of functional outcome after ICH. A recent meta‐analysis indicated a positive correlation between anemia and an increased risk of poor functional outcomes (pooled OR, 1.49; 95% CI, 1.17–1.89; *p* = 0.001).^35^ This relationship was supposed to be partly mediated by hematoma volume in several studies; a lower hemoglobin level resulted in abnormal coagulation, hemostatic alterations, and prolonged bleeding.^36^, ^37^ However, in our multivariate analysis, anemia had a greater impact than hematoma volume. Similar results were also reported by Kuramatsu et al.,^37^ who showed that the relevance of anemia was even more striking in patients with minor‐volume ICH than in all patients. This suggests that anemia itself may lead to cerebral injury with neuronal tissue hypoxia, metabolic distress, and cell energy dysfunction due to a reduction in oxygen‐carrying capacity.^38^, ^39^

By applying the aforementioned factors, we constructed a novel nomogram model to predict the post‐ICH outcome status. This model showed great predictive value, with AUC‐ROC of 0.842 and 0.831 in the training and validation sets, respectively. Moreover, we recognized the superior prognostic utility of this nomogram, as compared to that of the ICH score. The undesirable discrimination of the ICH score might be attributed to two reasons: first, the patients in our study were less severe than those enrolled to develop the ICH score,^4^, ^40^ as evidenced by the higher GCS score, smaller hematoma volume, and fewer IVH extensions on admission. Second, the median age in our cohorts was 62 years; thus, concerns have been raised that converting such variables according to cutoff values of <80 and >80 years could possibly sacrifice prognostic information for the majority of our patients. In fact, previous studies showed that the ICH score had great discrimination against mortality; however, the results varied with respect to functional outcomes.^5^ Our nomogram may be more applicable to clinically stable patients and compensate for the shortcomings of the ICH score.

As a multifactor graphical predictive tool, the advantage of a nomogram is that it can provide an individualized estimation for the prediction of the event of interest, which is entirely based on each included factor, without averaging or combining within a category.^41^ Hence, nomograms have been extensively used in cancer, surgery, and other specialties.

Our study has some limitations. First, this analysis was based on tertiary institutions, and those who underwent hematoma evacuation or were unstable to tolerate MRI were not included, which might have introduced the possibility of selection bias and limited the generalizability of our observations. Second, MRI scans were not collected serially in our cohorts, which makes it possible that DWILs had reversed or would present later by the time of undergoing MRI imaging. In fact, Menon et al.^13^ reported new ischemic lesions as late as 1 month from ictus. Third, MRI images were not routinely collected in some institutions, which could limit the utility and availability of our model. Finally, although our nomogram was externally validated and presented favorable discrimination and calibration, it should be noted that there were 23 outcomes only in the validation cohort. Considering the relatively small sample size, a multicenter validation study is required to confirm its performance.

In conclusion, the present study with a relatively large sample size provided further evidence that DWILs were associated with the 90‐day outcome in ICH. Based on this finding, we developed and externally validated the ANAID‐ICH nomogram to predict the probability of a 90‐day unfavorable outcome in patients with ICH. Our nomogram, which showed remarkable discrimination and calibration, would be helpful for making therapeutic decisions and conducting patient surveillance.

## AUTHOR CONTRIBUTIONS

FG and LT have full access to all data in the study and take responsibility for the integrity of the data and the accuracy of data analysis. JL and DL equally contributed to the study and were involved in data analysis and manuscript writing. JL, LT, and FG designed the study. LT and FG reviewed, edited, and approved the final version of the manuscript. FP, QK, HL, and MC collected the data.

## FUNDING INFORMATION

This study was funded by the National Natural Science Foundation of China (NSFC) (81471168, 81971155), the Science and Technology Action Plan for Major Diseases Prevention and Control in China (2017ZX‐01S‐006S3), and the Science and Technology Department of Zhejiang Province (2022KY174).

## CONFLICT OF INTEREST

None.

## Supporting information


Table S1

## Data Availability

The data that support the findings of this study are available from the corresponding author upon reasonable request.

## References

[1] Cordonnier C , Demchuk A , Ziai W , Anderson CS . Intracerebral haemorrhage: current approaches to acute management. Lancet. 2018;392(10154):1257‐1268. 30319113 10.1016/S0140-6736(18)31878-6

[2] van Asch CJ , Luitse MJ , Rinkel GJ , van der Tweel I , Algra A , Klijn CJ . Incidence, case fatality, and functional outcome of intracerebral haemorrhage over time, according to age, sex, and ethnic origin: a systematic review and meta‐analysis. Lancet Neurol. 2010;9(2):167‐176. 20056489 10.1016/S1474-4422(09)70340-0

[3] Li Q , Warren AD , Qureshi AI , et al. Ultra‐early blood pressure reduction attenuates hematoma growth and improves outcome in intracerebral hemorrhage. Ann Neurol. 2020;88(2):388‐395. 32453453 10.1002/ana.25793 PMC8697414

[4] Hemphill JC , Bonovich DC , Besmertis L , Manley GT , Johnston SC . The ICH score: a simple, reliable grading scale for intracerebral hemorrhage. Stroke. 2001;32(4):891‐897. 11283388 10.1161/01.str.32.4.891

[5] Gregório T , Pipa S , Cavaleiro P , et al. Assessment and comparison of the four Most extensively validated prognostic scales for intracerebral hemorrhage: systematic review with meta‐analysis. Neurocrit Care. 2019;30(2):449‐466. 30426449 10.1007/s12028-018-0633-6

[6] Parry‐Jones AR , Abid KA , Di Napoli M , et al. Accuracy and clinical usefulness of intracerebral hemorrhage grading scores: a direct comparison in a UKpopulation. Stroke. 2013;44(7):1840‐1845. 23686981 10.1161/STROKEAHA.113.001009

[7] Hwang DY , Dell CA , Sparks MJ , et al. Clinician judgment vs formal scales for predicting intracerebral hemorrhage outcomes. Neurology. 2016;86(2):126‐133. 26674335 10.1212/WNL.0000000000002266 PMC4731687

[8] Schmidt FA , Liotta EM , Prabhakaran S , Naidech AM , Maas MB . Assessment and comparison of the max‐ICH score and ICH score by external validation. Neurology. 2018;91(10):e939‐e946. 30068631 10.1212/WNL.0000000000006117 PMC6139815

[9] Lei C , Wu B , Liu M , Zhang S , Yuan R . Cerebral amyloid Angiopathy‐related intracerebral hemorrhage score for predicting outcome. Curr Neurovasc Res. 2016;13(2):156‐162. 26903393 10.2174/1567202613666160223122634

[10] Xu D , Gao Q , Wang F , et al. Sphingosine‐1‐phosphate receptor 3 is implicated in BBB injury via the CCL2‐CCR2 axis following acute intracerebral hemorrhage. CNS Neurosci Ther. 2021;27(6):674‐686. 33645008 10.1111/cns.13626 PMC8111497

[11] Song D , Ji YB , Huang XW , et al. Lithium attenuates blood‐brain barrier damage and brain edema following intracerebral hemorrhage via an endothelial Wnt/β‐catenin signaling‐dependent mechanism in mice. CNS Neurosci Ther. 2022;28(6):862‐872. 35343071 10.1111/cns.13832 PMC9062576

[12] Kimberly WT , Gilson A , Rost NS , et al. Silent ischemic infarcts are associated with hemorrhage burden in cerebral amyloid angiopathy. Neurology. 2009;72(14):1230‐1235. 19349602 10.1212/01.wnl.0000345666.83318.03 PMC2677484

[13] Menon RS , Burgess RE , Wing JJ , et al. Predictors of highly prevalent brain ischemia in intracerebral hemorrhage. Ann Neurol. 2012;71(2):199‐205. 22367992 10.1002/ana.22668 PMC3298034

[14] Kang DW , Han MK , Kim HJ , et al. New ischemic lesions coexisting with acute intracerebral hemorrhage. Neurology. 2012;79(9):848‐855. 22843271 10.1212/WNL.0b013e3182648a79

[15] Gregoire SM , Charidimou A , Gadapa N , et al. Acute ischaemic brain lesions in intracerebral haemorrhage: multicentre cross‐sectional magnetic resonance imaging study. Brain J Neurol. 2011;134(Pt 8):2376‐2386. 10.1093/brain/awr172 21841203

[16] Kidwell CS , Rosand J , Norato G , et al. Ischemic lesions, blood pressure dysregulation, and poor outcomes in intracerebral hemorrhage. Neurology. 2017;88(8):782‐788. 28122903 10.1212/WNL.0000000000003630 PMC5344081

[17] Wu B , Yao X , Lei C , Liu M , Selim MH . Enlarged perivascular spaces and small diffusion‐weighted lesions in intracerebral hemorrhage. Neurology. 2015;85(23):2045‐2052. 26546632 10.1212/WNL.0000000000002169 PMC4676754

[18] Xu X h , Ye X h , Li J w , et al. Association between remote diffusion‐weighted imaging lesions and cerebral small vessel disease in primary intracerebral hemorrhage. Eur J Neurol. 2019;26(7):961‐968. 30742740 10.1111/ene.13915

[19] Murthy SB , Cho SM , Gupta A , et al. A pooled analysis of diffusion‐weighted imaging lesions in patients with acute intracerebral hemorrhage. JAMA Neurol. 2020;77(11):1390. 32687564 10.1001/jamaneurol.2020.2349 PMC7372494

[20] Balachandran VP , Gonen M , Smith JJ , DeMatteo RP . Nomograms in oncology: more than meets the eye. Lancet Oncol. 2015;16(4):e173‐e180. 25846097 10.1016/S1470-2045(14)71116-7 PMC4465353

[21] Kim Y , Margonis GA , Prescott JD , et al. Nomograms to predict recurrence‐free and overall survival after curative resection of adrenocortical carcinoma. JAMA Surg. 2016;151(4):365‐373. 26676603 10.1001/jamasurg.2015.4516 PMC4967352

[22] Lopez A , Cacoub P , Macdougall IC , Peyrin‐Biroulet L . Iron deficiency anaemia. Lancet Lond Engl. 2016;387(10021):907‐916. 10.1016/S0140-6736(15)60865-0 26314490

[23] Vickers AJ , Elkin EB . Decision curve analysis: a novel method for evaluating prediction models. Med Decis Mak Int J Soc Med Decis Mak. 2006;26(6):565‐574. 10.1177/0272989X06295361 PMC2577036 17099194

[24] Garg RK , Liebling SM , Maas MB , Nemeth AJ , Russell EJ , Naidech AM . Blood pressure reduction, decreased diffusion on MRI, and outcomes after intracerebral hemorrhage. Stroke. 2012;43(1):67‐71. 21980211 10.1161/STROKEAHA.111.629493 PMC3246540

[25] Novakovic N , Wilseck ZM , Chenevert TL , et al. Assessing early erythrolysis and the relationship to perihematomal iron overload and white matter survival in human intracerebral hemorrhage. CNS Neurosci Ther. 2021;27(10):1118‐1126. 34145764 10.1111/cns.13693 PMC8446214

[26] Mader MM , Böger R , Appel D , et al. Intrathecal and systemic alterations of L‐arginine metabolism in patients after intracerebral hemorrhage. J Cereb Blood Flow Metab off J Int Soc Cereb Blood Flow Metab. 2021;41(8):1964‐1977. 10.1177/0271678X20983216 PMC8327100 33461409

[27] Wang J , Tang XQ , Xia M , et al. Iron chelation suppresses secondary bleeding after intracerebral hemorrhage in angiotensin II‐infused mice. CNS Neurosci Ther. 2021;27(11):1327‐1338. 34346561 10.1111/cns.13706 PMC8504530

[28] Schreiber S , Bueche CZ , Garz C , Braun H . Blood brain barrier breakdown as the starting point of cerebral small vessel disease? ‐ new insights from a rat model. Exp Transl Stroke Med. 2013;5(1):4. 23497521 10.1186/2040-7378-5-4 PMC3618264

[29] Rajani RM , Quick S , Ruigrok SR , et al. Reversal of endothelial dysfunction reduces white matter vulnerability in cerebral small vessel disease in rats. Sci Transl Med. 2018;10(448):eaam9507. 29973407 10.1126/scitranslmed.aam9507

[30] Rodrigues MA , Samarasekera N , Lerpiniere C , et al. Association between computed tomographic biomarkers of cerebral small vessel diseases and long‐term outcome after spontaneous intracerebral hemorrhage. Ann Neurol. 2021;89(2):266‐279. 33145789 10.1002/ana.25949 PMC7894327

[31] Lan X , Han X , Liu X , Wang J . Inflammatory responses after intracerebral hemorrhage: from cellular function to therapeutic targets. J Cereb Blood Flow Metab off J Int Soc Cereb Blood Flow Metab. 2019;39(1):184‐186. 10.1177/0271678X18805675 PMC6311675 30346222

[32] Shoamanesh A , Cassarly C , Morotti A , et al. Intensive blood pressure lowering and DWI lesions in intracerebral hemorrhage: exploratory analysis of the ATACH‐2 randomized trial. Neurocrit Care. 2021;36:71‐81. doi:10.1007/s12028-021-01254-9 34292474

[33] Imai T , Matsubara H , Hara H . Potential therapeutic effects of Nrf2 activators on intracranial hemorrhage. J Cereb Blood Flow Metab off J Int Soc Cereb Blood Flow Metab. 2021;41(7):1483‐1500. 10.1177/0271678X20984565 PMC8221764 33444090

[34] Poon MTC , Fonville AF , Al‐Shahi SR . Long‐term prognosis after intracerebral haemorrhage: systematic review and meta‐analysis. J Neurol Neurosurg Psychiatry. 2014;85(6):660‐667. 24262916 10.1136/jnnp-2013-306476

[35] Acosta JN , Leasure AC , Kuohn LR , et al. Admission hemoglobin levels are associated with functional outcome in spontaneous intracerebral hemorrhage. Crit Care Med. 2021;49(5):828‐837. 33591003 10.1097/CCM.0000000000004891 PMC8611893

[36] Roh DJ , Albers DJ , Magid‐Bernstein J , et al. Low hemoglobin and hematoma expansion after intracerebral hemorrhage. Neurology. 2019;93(4):e372‐e380. 31209179 10.1212/WNL.0000000000007820 PMC6669932

[37] Kuramatsu JB , Gerner ST , Lücking H , et al. Anemia is an independent prognostic factor in intracerebral hemorrhage: an observational cohort study. Crit Care Lond Engl. 2013;17(4):R148. 10.1186/cc12827 PMC4057052 23880122

[38] Oddo M , Milby A , Chen I , et al. Hemoglobin concentration and cerebral metabolism in patients with aneurysmal subarachnoid hemorrhage. Stroke. 2009;40(4):1275‐1281. 19265059 10.1161/STROKEAHA.108.527911

[39] Lelubre C , Bouzat P , Crippa IA , Taccone FS . Anemia management after acute brain injury. Crit Care Lond Engl. 2016;20(1):152. 10.1186/s13054-016-1321-6 PMC4911680 27311626

[40] Hemphill JC , Farrant M , Neill TA . Prospective validation of the ICH score for 12‐month functional outcome. Neurology. 2009;73(14):1088‐1094. 19726752 10.1212/WNL.0b013e3181b8b332 PMC2764394

[41] Cappellari M , Mangiafico S , Saia V , et al. IER‐SICH nomogram to predict symptomatic intracerebral hemorrhage after Thrombectomy for stroke. Stroke. 2019;50(4):909‐916. 31233386 10.1161/STROKEAHA.118.023316

