# Supplementary material for: ANAID‐ICH nomogram for predicting unfavorable outcome after intracerebral hemorrhage
Source: CNS Neurosci Ther. 2022 Aug 24;28(12):2066–75. doi: 10.1111/cns.13941 (PMC9627367; doi:10.1111/cns.13941)
Supplement: Supplementary file 1 — Table S1 [file CNS-28-2066-s001.docx]

**Supplementary Table 1. Demographics and clinical characteristics of the training cohort and validation cohort**

| **Characteristic** | **Training Cohort**  **N = 750** | **Validation Cohort**  **N = 149** | ***p*-value** |
| --- | --- | --- | --- |
| **Age, y, median (IQR)** | 62 (52, 70) | 56 (49, 65) | **<0.001**** |
| **Male gender, n(%)** | 500 (66.67%) | 92 (61.74%) | 0.247 |
| **Medical history, n(%)** | | | |
| **Hypertension** | 574 (76.53%) | 101.00 (67.79%) | **0.024*** |
| **Diabetes mellitus** | 130.00 (17.33%) | 15 (10.07%) | **0.028*** |
| **Atrial fibrillation** | 25 (3.33%) | 0 (0.00%) | **0.024*** |
| **Prior ICH** | 51 (6.80%) | 4 (2.68%) | 0.056 |
| **Prior AIS/TIA** | 66 (8.80%) | 8 (5.37%) | 0.164 |
| **Antiplatelet** | 69 (9.20%) | 4 (2.68%) | **0.008*** |
| **Anticoagulation** | 11 (1.47%) | 0 (0.00%) | 0.227 |
| **Baseline clinical assessment, median (IQR)** | | | |
| **Onset to emergency, d** | 0.50 (0.25, 1.00) | 0.12 (0.08, 0.25) | **<0.001**** |
| **GCS score** | 15 (14, 15) | 15 (13, 15) | 0.050 |
| **NIHSS score** | 4 (2, 10) | 4 (2, 9) | 0.891 |
| **SBP, mmHg** | 159 (144, 178) | 180 (160, 195) | **<0.001**** |
| **DBP, mmHg** | 91 (80, 101) | 98 (87, 107) | **<0.001**** |
| **MAP, mmHg** | 114 (103, 125) | 126 (112, 136) | **<0.001**** |
| **Laboratory values, median (IQR)** | | | |
| **Hemoglobin, g/L** | 142 (130, 151) | 142 (134, 153) | 0.158 |
| **Anemia, n(%)** | 132 (17.60%) | 20 (13.42%) | 0.214 |
| **Platelet, 10^9^/L** | 197 (161, 237) | 198 (171, 240) | 0.596 |
| **INR** | 1.00 (0.96, 1.05) | 1.01 (0.97, 1.05) | 0.205 |
| **FBG, mmol/L** | 5.71 (5.02, 6.80) | 6.61 (5.86, 8.05) | **<0.001**** |
| **Creatine, umol/L** | 61 (51, 73) | 60 (50, 71) | 0.344 |
| **LDL ≤ 1.8, n(%)** | 133 (17.92%) | 22 (14.77%) | 0.353 |
| **Radiological variables** | | | |
| **ICH volume, ml, median (IQR)** | 9.00 (3.36, 17.78) | 9.17 (3.55, 17.17) | 0.919 |
| **ICH location, n(%)** |  |  |  |
| **Lobar, n(%)** | 167 (22.27%) | 16 (10.74%) | **0.001**** |
| **Deep, n(%)** | 498 (66.40%) | 111 (74.50%) | 0.053 |
| **Infratentorial, n(%)** | 110 (14.67%) | 22 (14.77%) | 0.975 |
| **IVH, n(%)** | 215 (28.67%) | 35 (23.49%) | 0.198 |
| **Onset to MRI, d, median (IQR)** | 6 (4, 7) | 10 (7, 15) | **<0.001**** |
| **Presence of DWILs, n(%)** | 131 (17.47%) | 26 (17.45%) | 0.996 |
| **90-day mRS 4-6, n(%)** | 153 (20.73%) | 23 (15.54%) | 0.149 |

**Abbreviations: ICH = intracerebral hemorrhage; AIS = acute ischemic stroke; TIA = transient ischemic attack; GCS = Glasgow coma scale; NIHSS = National Institutes of Health Stroke Scale; SBP = systolic blood pressure; DBP = diastolic blood pressure; MAP = mean arterial pressure; INR = international normalized ratio; FBG = fasting blood glucose; LDL = low-density lipoprotein; IVH = intraventricular extension hemorrhage; DWILs = diffusion-weighted imaging lesions; IQR= interquartile range.**

**p*<0.05

***p*<0.01
